# Adoptive transfer of M2 macrophages reduces neuropathic pain via opioid peptides

**DOI:** 10.1186/s12974-016-0735-z

**Published:** 2016-10-07

**Authors:** Maria Pannell, Dominika Labuz, Melih Ö. Celik, Jacqueline Keye, Arvind Batra, Britta Siegmund, Halina Machelska

**Affiliations:** 1 Department of Anesthesiology and Critical Care Medicine, Charité-Universitätsmedizin Berlin, Campus Benjamin Franklin, Hindenburgdamm 30, 12203 Berlin, Germany; 2 Medizinische Klinik für Gastroenterologie, Infektiologie und Rheumatologie, Charité-Universitätsmedizin Berlin, Campus Benjamin Franklin, Hindenburgdamm 30, 12203 Berlin, Germany

**Keywords:** Macrophage, Neuropathic pain, Enkephalin, Dynorphin, Endorphin, Analgesia

## Abstract

**Background:**

During the inflammation which occurs following nerve damage, macrophages are recruited to the site of injury. Phenotypic diversity is a hallmark of the macrophage lineage and includes pro-inflammatory M1 and anti-inflammatory M2 populations. Our aim in this study was to investigate the ability of polarized M0, M1, and M2 macrophages to secrete opioid peptides and to examine their relative contribution to the modulation of neuropathic pain.

**Methods:**

Mouse bone marrow-derived cells were cultured as unstimulated M0 macrophages or were stimulated into an M1 phenotype using lipopolysaccharide and interferon-γ or into an M2 phenotype using interleukin-4. The macrophage phenotypes were verified using flow cytometry for surface marker analysis and cytokine bead array for cytokine profile assessment. Opioid peptide levels were measured by radioimmunoassay and enzyme immunoassay. As a model of neuropathic pain, a chronic constriction injury (CCI) of the sciatic nerve was employed. Polarized M0, M1, and M2 macrophages (5 × 10^5^ cells) were injected perineurally twice, on days 14 and 15 following CCI or sham surgery. Mechanical and heat sensitivity were measured using the von Frey and Hargreaves tests, respectively. To track the injected macrophages, we also transferred fluorescently stained polarized cells and analyzed the surface marker profile of endogenous and injected cells in the nerves ex vivo.

**Results:**

Compared to M0 and M1 cells, M2 macrophages contained and released higher amounts of opioid peptides, including Met-enkephalin, dynorphin A (1–17), and β-endorphin. M2 cells transferred perineurally at the nerve injury site reduced mechanical, but not heat hypersensitivity following the second injection. The analgesic effect was reversed by the perineurally applied opioid receptor antagonist naloxone methiodide. M2 cells did not affect sensitivity following sham surgery. Neither M0 nor M1 cells altered mechanical and heat sensitivity in CCI or sham-operated animals. Tracing the fluorescently labeled M0, M1, and M2 cells ex vivo showed that they remained in the nerve and preserved their phenotype.

**Conclusions:**

Perineural transplantation of M2 macrophages resulted in opioid-mediated amelioration of neuropathy-induced mechanical hypersensitivity, while M1 macrophages did not exacerbate pain. Therefore, rather than focusing on macrophage-induced pain generation, promoting opioid-mediated M2 actions may be more relevant for pain control.

## Background

Around 7 % of Europeans suffer from chronic neuropathic pain [1]. This is characterized as a spontaneously occurring, burning pain with increased sensitivity to mechanical and thermal stimuli [2, 3]. It often develops following peripheral nerve injury as a result of trauma or disease such as herpes virus or diabetes mellitus, and only 40–60 % of sufferers gain relief from treatment [2, 4]. The systemic administration of classical opioids such as morphine might be an effective treatment in some neuropathic conditions [5]. The use of these drugs remains controversial, however, due to the high dosage required and the central side effects, including sedation, respiratory depression, cognitive impairment, and dependence [6, 7]. The obvious and numerous drawbacks of the current treatment highlight the need for new, more effective therapeutic strategies and mechanistic insights into neuropathic pain control. There is evidence that activation of opioid receptors on peripheral sensory neurons results in effective analgesia devoid of central adverse effects. This can be achieved through the injection of exogenous opioids in small, systemically inactive doses into peripheral injured tissue or via endogenous opioid peptides, including Met-enkephalin, dynorphin A (1-17) (dynorphin), and β-endorphin released from locally accumulating immune cells [8–11].

During the inflammation which occurs as a result of nerve injury, macrophages are recruited to the site of damage [12–14]. Since the leukocyte influx coincides with pain, earlier studies focused on pro-nociceptive actions of macrophages. Strategies based on general depletion of these cells resulted in either complete blockade, moderate reduction, or no changes in neuropathy-induced hypersensitivity [15–18]. Importantly, macrophages are characterized by phenotypic diversity, including pro-inflammatory M1 and anti-inflammatory M2 populations [19, 20]. In previous studies, the macrophage function was modified by using agonists of the peroxisome proliferator-activated receptor-γ (PPAR-γ), which promote the polarization toward the M2 phenotype [20, 21]. Application of a PPAR-γ agonist into incised paws increased M2 macrophage numbers and mRNA levels of their markers, including the anti-inflammatory cytokine interleukin-10 (IL-10), which was associated with the attenuation of postoperative pain. This analgesic effect was mimicked by a local transfer of PPAR-γ agonist-treated peritoneal macrophages [22]. Additionally, PPAR-γ or toll-like receptor 4 activation in inflamed paws elevated nociceptive thresholds, which was reversed by local application of an opioid receptor antagonist, indicating the involvement of endogenous opioid peptides in the carrageenan or complete Freund’s adjuvant (CFA) inflammatory pain models [23–25].

The role of polarized macrophages in neuropathic pain has not been as extensively studied. Injured nerves following partial sciatic nerve ligation were found to be infiltrated by M1 and M2 macrophages [26], and perineural transplantation of the PPAR-γ agonist-treated macrophages attenuated neuropathy-induced mechanical hypersensitivity [27]. Another study found that injection of IL-4 at the nerve injury site shifted the macrophage phenotype to the M2 anti-inflammatory state, which was associated with decreased mRNA levels of pro-inflammatory cytokines and elevated mRNA levels of anti-inflammatory cytokines, and correlated with amelioration of tactile and thermal hypersensitivity [28]. Together, studies examining the role of phenotypically different macrophage populations in neuropathy are sparse and exclusively focused on the actions of macrophage-derived cytokines. Notably, immune cells accumulating at damaged nerves also contain opioid peptides, which upon release activate local neuronal opioid receptors and attenuate neuropathy-induced hypersensitivity in animal models [29–32]. However, the potential role of opioid peptides derived from different macrophage populations in neuropathy has so far not been assessed.

In this study, we investigated the relative contribution of M0, M1, and M2 macrophages to the modulation of neuropathic pain using an adoptive transfer strategy, in a chronic constriction injury (CCI) mouse model. To this end, we cultured bone marrow-derived macrophages from mice, inducing a pro-inflammatory M1 phenotype using lipopolysaccharide (LPS) and interferon-γ (IFN-γ), and an anti-inflammatory M2 phenotype using IL-4. The unstimulated M0 phenotype represented a low activation state. Surface marker analysis by flow cytometry and cytokine release by cytokine bead array (CBA) were used to distinguish activation phenotypes. The opioid peptide content and release were measured using radioimmunoassay (RIA) and enzyme immunoassay (EIA). Finally, the relevance of polarized macrophages in modulation of mechanical and heat sensitivity in vivo was examined using von Frey and Hargreaves tests, respectively.

## Methods

### Animals

Experiments were approved by the State animal care committee (Landesamt für Gesundheit und Soziales, Berlin, Germany) and were performed according to the Guide for the Care and Use of Laboratory Animals adopted by the U.S. National Institutes of Health and the ARRIVE guidelines [33]. Male C57BL/6J mice (22–30 g, 6–13 weeks old; Janvier Laboratories, France; bred at the Charité-Campus Benjamin Franklin, Berlin, Germany) were kept in groups of three to five per cage, with free access to food and water, in environmentally controlled conditions (12 h light/dark schedule, light on at 0700 h; 22 ± 0.5 °C; humidity 60–65 %). Animals were randomly placed in cages by an animal caretaker who was not involved in the study. In vivo experiments were performed by an experimenter blinded to the treatments. Macrophages and substances were prepared in separate, coded vials by a colleague not involved in in vivo testing. The codes were broken after completion of the experiment. After completion of in vivo experiments and for tissue collection for ex vivo experiments, animals were killed with isoflurane-overdose (AbbVie). All efforts were made to minimize animal suffering and to reduce the number of animals used.

### Culture and polarization of bone marrow-derived macrophages

Bone marrow-derived cells were isolated and cultured as previously described [34]. In summary, mice were killed by isoflurane overdose and the femurs and tibias were removed, keeping the femoral head and femur intact, and cutting off the paw bones below the distal end of the tibia. Any muscle connected to the bone was carefully removed. Bones were stored in sterile Hanks’ balanced salt solution (HBSS) on ice until all bones had been collected. After cutting through the epiphysis at both ends of the femur and tibia, sterile HBSS was slowly flushed through the bone using a 23-G needle and 5-ml syringe, and the content was collected in a sterile 15-ml polypropylene tube. Cells were dissociated by briefly passing them through the syringe, followed by straining using a 70-μm cell strainer. After centrifugation (4 °C, 7 min at 500*g*), red blood cells were lysed by adding 1 ml erythrocyte lysis buffer (QIAGEN). After 1–2 min, HBSS (20 ml) was added, the suspension was centrifuged as before, and the supernatant was removed. Cells were washed in complete Dulbecco’s modified Eagle medium (DMEM; 20 ml) and again centrifuged. After removal of the supernatant, cells were plated in a 10-cm dish with macrophage colony-stimulating factor (MCSF; 10 ng/ml; PeproTech) to allow differentiation of bone marrow cells to macrophages. After 2 days, 5 ml of media was exchanged from each dish with MCSF (10 ng/ml). After 6 days, 5 ml of media was exchanged from each dish without an addition of MCSF. For polarization of cells to an M2 phenotype, cells were stimulated with IL-4 (20 ng/ml; BD Biosciences) on day 6 for 48 h. For the M1 phenotype, cells were stimulated on day 7 with LPS (100 ng/ml; InvioGen) and IFN-γ (20 ng/ml; PeproTech) for 24 h. The M0 phenotype represents unstimulated cells and were therefore only subjected to the media change on day 6 [35–37].

### Flow cytometry analysis of cell surface markers

To determine the expression of surface markers indicating macrophage polarization status, cultured bone marrow macrophages were first labeled with LIVE/DEAD® fixable aqua dead cell stain kit, for 30 min on ice, according to the manufacturer’s instructions (ThermoFisher Scientific), to exclude dead cells. Cells were then washed with ice-cold FACS buffer (0.5 % BSA/PBS), followed by centrifugation and removal of the supernatant. Cells were then stained for 20 min on ice with anti-mouse F4/80-eFluor 450 antibody (0.2 μg/100 μl; eBioscience) as a general marker of macrophages, anti-mouse major histocompatibility complex II (MHC II)-PE-Cy7 antibody (0.1 μg/100 μl; eBioscience) as a marker of M1 macrophages, and mouse anti-rat cluster of differentiation 163 (CD163)-Alexa 647 antibody (1 μg/100 μl; AbD Serotec) as a marker of M2 macrophages. All antibodies were prepared in FACS buffer. The staining specificity of antibodies was tested by using fluorescence minus one (FMO) controls [38]. The percentages of positively stained cells determined over 10,000 events were analyzed using FACS Canto II (BD Biosciences), and fluorescence intensity was expressed in arbitrary units on a logarithmic scale and analyzed using FlowJo software (version 10.1r5; TreeStar, Inc.) [39].

### Measurement of cytokine levels

Following polarization, macrophages were washed and seeded in 24-well plates at 2 × 10^5^ cells per well in 500 μl complete DMEM. Approximately 18 h after seeding, media was collected and frozen at −20 °C for analysis of released cytokines. The concentration of cytokines was measured using CBA (BD Biosciences), allowing precise analysis of six cytokines, including IL-6, IL-10, IL-12p70, monocyte chemoattractant protein-1 (MCP-1), IFN-γ, and tumor necrosis factor-α (TNF-α), as per manufacturer’s instructions. CBA samples were run on a FACS Canto II cytometer (BD Biosciences). Data were analyzed using FCAP-array software (version 3.1; BD Biosciences) to convert fluorescent intensity values to concentrations using a 10-point standard curve (0–5000 pg/ml) as described previously [40].

### Measurement of opioid peptide levels

Following polarization, macrophages were washed and seeded at 5 × 10^5^ cells per well in a 24-well plate with 650 μl complete DMEM. Approximately 18 h after seeding, media was removed and frozen at −20 °C for later analysis of secreted levels of opioid peptides, Met-enkephalin, dynorphin, and β-endorphin. To measure the intracellular opioid peptide content, the cells were scraped from each well, resuspended in RPMI buffer, and lysed by a freezing/thawing procedure (8 min at −80 °C and 1 min at 50 °C; repeated five times) followed by sonication (Ultra-Turrax T8; IKA Labortechnik) [29].

Immunoreactivities of Met-enkephalin and dynorphin were determined using RIA kits (Peninsula Laboratories), while those of β-endorphin were assessed using EIA kits (Phoenix Pharmaceuticals), according to manufacturers’ instructions and previous studies [29, 41]. Briefly, for Met-enkephalin and dynorphin measurements using RIA, the samples were thawed and incubated overnight (4 °C) with anti-Met-enkephalin or anti-dynorphin, respectively. Respective ^125^I-labeled opioid peptide tracers were then added and the samples were incubated overnight at 4 °C. The following day, goat anti-rabbit IgG and normal rabbit serum were added and the samples were incubated for 90 min at room temperature. RIA buffer was then applied, samples were centrifuged, and radioactivity levels were measured in the pellets using a gamma counter (Wallac/PerkinElmer 1470 Wizard), according to the standard curve.

For EIA β-endorphin measurements, samples were thawed and incubated with anti-β-endorphin and biotinylated peptide (2 h), followed by addition of streptavidin-horseradish peroxidase (1 h). After washing, tetramethylbenzidine was added (1 h), the reaction was terminated by application of 2 N HCl (1 h), and the absorbance was measured at 450 nm (Molecular Devices Spectra Max), according to the standard curve.

All RIA and EIA samples were measured in duplicates, in two to three independent assays [29]. In earlier studies, we verified the specificity of opioid peptide antibodies by testing all three standard opioid peptides (Met-enkephalin, dynorphin, and β-endorphin) in all three RIAs and showing that each opioid peptide was only recognized by its respective antibody [41], and by showing the absence of opioid peptide immunoreactivities in immune cells from the corresponding opioid peptide knockout mice [29].

### Chronic constriction injury

CCI was induced in deeply isoflurane-anesthetized mice by exposing the sciatic nerve at the level of the right mid-thigh and placing three loose silk ligatures (4/0) around the nerve with about 1-mm spacing; the ligatures were tied until they elicited a brief twitch in the respective hind limb. Sham operation was performed in a similar manner but without nerve ligation. The wound was closed with silk sutures [30].

### Evaluation of mechanical sensitivity (von Frey test)

Animals were habituated to the test cages daily (one to two times for 15 min), starting 6 days prior to nociceptive testing; they were individually placed in clear plexiglas cubicles located on a stand with anodized mesh (Model 410; IITC Life Sciences). To assess the sensitivity, calibrated von Frey filaments in the range of 0.054 mN (0.0056 g) to 42.85 mN (4.37 g) were used (Stoelting). The filaments were applied until they bowed, for approximately 3 s, to the plantar surface of hind paws. The up-down method was used to estimate 50 % withdrawal thresholds [42].Testing began using a 2.74-mN (0.28 g) filament. If the animal withdrew the paw, the preceding weaker filament was applied. In the absence of withdrawal, the next stronger filament was applied. The maximum number of applications was 6–9, and the cutoff was 42.85 mN (4.37 g). The sequence of paws was alternated between animals to avoid “order” effects, according to our previous studies [30, 43, 44].

### Evaluation of heat sensitivity (Hargreaves test)

Before experiments, mice were habituated as described for the von Frey test, except they were placed in clear plexiglas chambers positioned on a stand with a glass surface (Model 336; IITC Life Sciences). To examine heat sensitivity, radiant heat was applied to the plantar surface of hind paws from underneath the glass floor with a high-intensity projector lamp bulb and paw withdrawal latency was evaluated using an electronic timer. The withdrawal latency was defined as the average of two measurements separated by at least 10 s. The heat intensity was adjusted to obtain baseline withdrawal latency of about 10–12 s in uninjured paws, and the cutoff was set at 20 s to avoid tissue damage [44, 45].

### Assessment of analgesic effects of polarized macrophages

Freshly prepared polarized M0, M1, or M2 macrophages (5 × 10^5^ in 30 μl DMEM) were injected perineurally at the site of nerve injury (CCI site) twice, on days 14 and 15 following CCI. Mechanical and heat sensitivities were measured just before CCI, and then after CCI on day 14 (before and 1 and 5 h after the first injection), on day 15 (before and 1 and 5 h after the second injection), and then daily on days 16–19. Control groups were treated with DMEM (30 μl) only. Analogous experiments were performed in sham-operated mice. The concentration of 5 × 10^5^ cells was the most effective of 2.5–10 × 10^5^ cells tested in pilot experiments using M2 macrophages.

To assess the contribution of peripheral (at the CCI site) opioid receptors to M2 macrophage-induced analgesia, a peripherally restricted opioid receptor antagonist, naloxone methiodide (NLXM; Sigma-Aldrich) was used. NLXM (10 μg/30 μl 0.9 % NaCl) was injected at the CCI site 24 h after the second injection of M2 macrophages (i.e., on day 16 after CCI). Mechanical sensitivity was measured before NLXM injection (on days 14 and 15 after CCI) and after NLXM injection, 15 min and 1–5 h (on day 16 after CCI), and 24 h (on day 17 after CCI). The control group was treated with 0.9 % NaCl (30 μl).

For perineural injections, performed under brief isoflurane anesthesia, a polyethylene tube was placed 2 mm from the tip around the needle (attached to a Hamilton syringe; Sigma-Aldrich) to ensure the same depth of needle insertion into the middle of the scar after surgeries [29, 30].

### Detection of transferred macrophages in injured nerves

To track the cultured macrophages following in vivo perineural injections, M0, M1, and M2 macrophages were treated immediately following polarization with carboxyfluorescein succinimidyl ester (CFSE; 0.5 μM; Sigma) for 5 min at 37 °C. The cells were then quenched using DMEM, followed by centrifugation and a further two washes with DMEM alone [46].

Fluorescently labeled M0, M1, and M2 macrophages (each at 5 × 10^5^ in 30 μl DMEM) or DMEM alone (30 μl) were injected at the CCI site on day 14 (for one injection) or on days 14 and 15 (for two injections) after CCI, analogously to unlabeled macrophages described above. Mice were killed 24 h after the first injection and 24 h after the second injection, and injured nerves (approximately 1-cm long, including the ligation site and sites distal and proximal to it) were removed. Nerves were cut into small pieces and collected in a digestive solution (10 ml RPMI1640 with GlutaMax, 0.5 ml HEPES, 30 mg collagenase, 10 mg hyaluronidase, and 2 % FBS) for 30 min at 37 °C. Afterward, the tissue was filtered through a 70-μm pore sieve (BD Biosciences), washed with RPMI media, and centrifuged and cell pellets were resuspended in RPMI media. The cells were counted and verified for viability in a Neubauer chamber (Optik Labor) using the trypan blue exclusion method, as described previously [29, 30].

For analysis of CFSE labeling, the cells isolated from nerves were stained with LIVE/DEAD® fixable aqua dead cell stain kit, anti-mouse CD45-APC-Alexa780 antibody (0.1 μg/100 μl; eBioscience) to gate out non-hematopoietic cells and debris, and with antibodies to macrophage markers, anti-F4/80-eFluor 450, anti-MHC II-PE-Cy7, and anti-CD163-Alexa 647, as described above. The CFSE staining was analyzed in the FITC channel. To determine the absolute cell numbers, CountBright™ Absolute Counting Beads (ThermoFisher Scientific) were used. The absolute cell numbers and the percentage of positively stained cells were determined over 20,000 events, and the data were analyzed using FlowJo software, as specified above.

### Statistical analyses

Statistical analyses were performed using GraphPad Prism software (Version 5.02 for Windows; GraphPad Software Inc.). Data are expressed as means ± SEM. One-way repeated measures (RM) analysis of variance (ANOVA) was used to analyze more than two groups for dependent data, while one-way ANOVA was used to analyze more than two groups for independent data. Two-way RM ANOVA was applied to compare two groups over time (more than two time points). In all cases, Bonferroni’s multiple comparison test was used as a post hoc test. An unpaired two-tailed *t* test was used to compare two groups for independent data. Differences were considered significant at values of *p* < 0.05.

## Results

### Polarized bone marrow-derived macrophages display surface marker and cytokine profiles for M1 and M2 activation

**Fig. 1:**
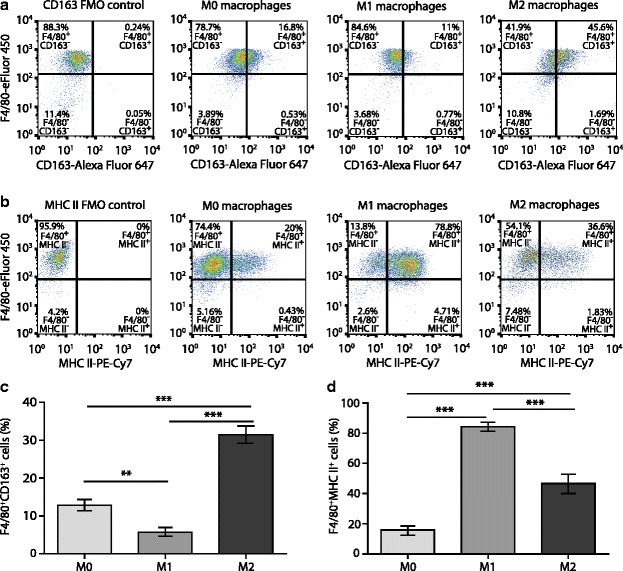
Analysis of surface markers on cultured macrophages following polarization. **a** Representative *dot blots* showing cell populations expressing F4/80 and CD163. The *blots* represent the CD163 FMO-negative control (*far left*), M0-polarized cells (*second left*), M1 cells (*third left*), and M2 cells (*far right*). The percentage of macrophages negative for CD163 (F4/80^+^CD163^−^) are shown in top left quadrants, while macrophages expressing CD163 (F4/80^+^CD163^+^) are shown in top right quadrants. **b** Representative *dot blots* showing cell populations expressing F4/80 and MHC II. The blots show the MHC II FMO-negative control (*far left*), the M0 cells (*second left*), the M1 cells (*third left*), and the M2 cells (*far right*). The percentage of macrophages negative for MHC II (F4/80^+^MHC II^−^) are shown in *top left quadrants*, while macrophages expressing MHC II (F4/80^+^MHC II^+^) are shown in *top right quadrants*. **c** Percentage of polarized M0, M1, and M2 macrophages expressing CD163. **d** Percentage of polarized M0, M1, and M2 macrophages expressing MHC II. The data were analyzed using flow cytometry and FlowJo software. In **c** and **d**, **p < 0.01, ***p < 0.001 (one-way RM ANOVA, Bonferroni’s test). Data show the mean ± SEM (*n* = 8 samples per group)

Figure 1a, b depicts representative flow cytometry dot blots from M0, M1, and M2 polarized macrophages to show that the majority of analyzed cells were positive for the macrophage marker F4/80, demonstrating successful generation of macrophages from the bone marrow cells. To distinguish the macrophage phenotypes, we analyzed the surface markers MHC II and CD163. MHC II is expressed on antigen presenting cells and is upregulated during inflammation [47, 48]; it is therefore commonly used as a marker for the M1 phenotype [49]. CD163 is a scavenger receptor for the hemoglobin-haptoglobin complex [50] and is a marker of cells from the macrophage-monocyte lineage [51]. It has commonly been used as a marker of M2 activation [52]. The analysis of CD163 expression revealed that the percentage of CD163^+^ M2 cells was significantly higher than both M0 and M1 cells. Additionally, the percentage of CD163^+^ M1 cells was significantly lower compared to M0 cells (Fig. 1a, c). The analysis of MHC II expression showed that the percentage of MHC II^+^ M1 cells was significantly higher than that of M0 and M2 cells. There was also higher percentage of MHC II^+^ M2 cells compared to that of M0 cells (Fig. 1b, d). The staining specificity of CD163 and MHC II antibodies was confirmed by minimal CD163 staining (0.24 %) (Fig. 1a) or absence of MHC II staining (0 %) in FMO controls (Fig. 1b).

**Fig. 2:**
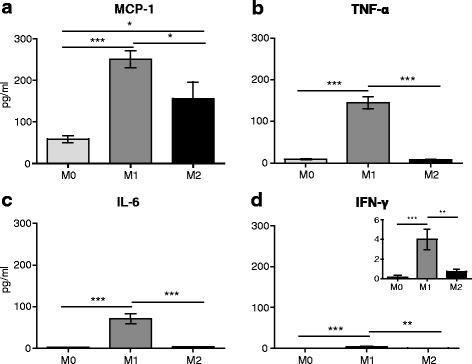
Pro-inflammatory cytokine release profiles of cultured macrophages following polarization. Released levels of MCP-1 (**a**), TNF-α (**b**), IL-6 (**c**), and IFN-γ (**d**). The insert in **d** shows the graph with smaller *Y*-axis scale since IFN-γ levels were very low; only levels for M1 were above detection limit. The cytokine concentrations were measured in media using CBA, ~18 h after polarization was completed. *p < 0.05, **p < 0.01, ***p < 0.001 (one-way RM ANOVA, Bonferroni’s test). Data show the mean ± SEM (*n* = 8 samples per group)

Cytokine release profile is another good indicator of cell polarization, with M1-activated macrophages commonly releasing pro-inflammatory cytokines. Accordingly, analysis using CBA showed significantly higher released levels of MCP-1, TNF-α, IL-6, and IFN-γ from M1 polarized cells compared to M0 and M2 cells. MCP-1 was also secreted in higher amounts by M2 versus M0 cells, but there were no differences in the secretion of TNF-α, IL-6, and IFN-γ between M2 and M0 cells (Fig. 2a–d). The levels of IL12p70 and of the anti-inflammatory cytokine IL-10 were below the detection limit and therefore not shown. Together, the analysis of cell surface markers and cytokine profile support the polarization of bone marrow-derived macrophages into an M1 phenotype expressing MHC II and releasing pro-inflammatory cytokines and an M2 phenotype expressing CD163 and secreting minimal levels of pro-inflammatory cytokines.

### M2 polarized macrophages contain and release more opioid peptides than M0 and M1 cells

**Fig. 3:**
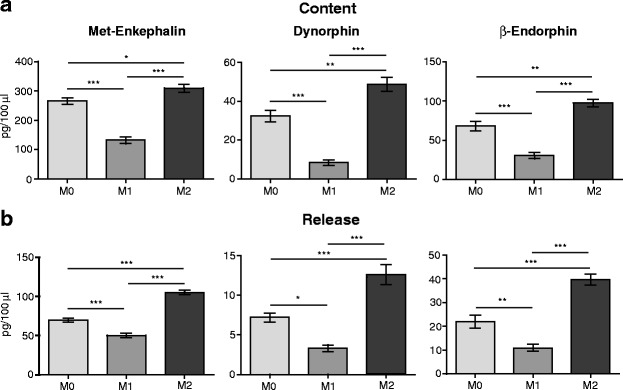
Opioid peptide content and release from cultured macrophages following polarization. Intracellular content (**a**) and extracellular, released levels (**b**) of Met-enkephalin, dynorphin, and β-endorphin. Opioid peptide levels were measured by RIA (Met-enkephalin and dynorphin) or EIA (β-endorphin), ~18 h after polarization was completed. *p < 0.05, **p < 0.01, ***p < 0.001 (one-way RM ANOVA, Bonferroni’s test). Data show the mean ± SEM (*n* = 8 samples per group)

Since opioid peptides have so far not been comprehensively analyzed in macrophage subpopulations, we measured the content and release of Met-enkephalin, dynorphin, and β-endorphin from polarized macrophages. We found that M2-polarized cells contained (Fig. 3a) and secreted (Fig. 3b) significantly higher levels of all three opioid peptides than M1 and M0 macrophages. Additionally, M1 macrophages contained and released significantly lower opioid peptide levels that M0 cells (Fig. 3a, b).

### Adoptive transfer of M2, but not M1 and M0, macrophages at the injured nerve results in analgesia

**Fig. 4:**
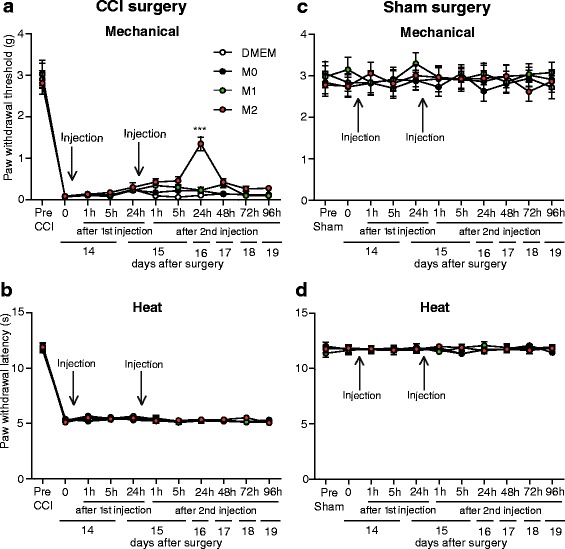
Effects of polarized macrophages transferred at the nerves on sensitivity following CCI and sham surgery. **a**, **b** Effects of M0, M1, and M2 macrophages on mechanical (**a**) and heat hypersensitivity (**b**) following CCI. **c**, **d** Effects of M0, M1, and M2 macrophages on mechanical (**c**) and heat sensitivity (**d**) following sham surgery. Macrophages or control DMEM were injected at the site of CCI or sham surgery on days 14 and 15 following surgeries (indicated by *arrows*). Mechanical and heat sensitivity were measured using von Frey filaments and Hargreaves test, respectively. ***p < 0.001 vs. control (DMEM); two-way RM ANOVA, Bonferroni’s test. Data show the mean ± SEM (*n* = 9 animals per group)

**Fig. 5:**
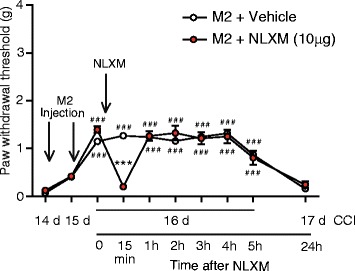
Contribution of peripheral opioid receptors to M2 macrophage-mediated analgesia in neuropathy-induced mechanical hypersensitivity. M2 macrophages were injected on days 14 and 15 following CCI, and opioid receptor antagonist NLXM was applied on day 16 after CCI (i.e., 24 h after the second injection of macrophages). All injections (indicated by *arrows*) were performed at the CCI site, and the effects were assessed using von Frey filaments. ***p < 0.001 vs. vehicle; ^###^ *p* < 0.001 vs. baseline measured 14 days after CCI (two-way RM ANOVA, Bonferroni’s test). Data show the mean ± SEM (*n* = 6 animals per group)

To access the effects of polarized macrophages on neuropathy-induced mechanical and heat hypersensitivity, we injected 5 × 10^5^ of M0-, M1-, or M2-polarized macrophages at the nerve injury site 14 days following CCI. Cells were injected directly after polarization and were washed twice to remove residual polarizing agents. In parallel, excess cells were seeded to analyze cell surface markers and opioid and cytokine levels (results discussed above). Mechanical and heat sensitivity were measured using the von Frey test and Hargreaves test respectively, at 1, 5, and 24 h after injection. We found that up to 24 h following the first injection, none of the injected macrophage populations had an effect on mechanical hypersensitivity (Fig. 4a). We then injected polarized cells (5 × 10^5^) a second time, following the 24-h measurement after the first injection (i.e., on day 15 after CCI). We found that the second M2 cell injection elevated mechanical thresholds 24 h later (Fig. 4a). This analgesic effect was maintained for at least 29 h (Fig. 5) and resolved 48 h after the second injection (i.e., on day 17 after CCI) (Figs. 4a and 5). The application of M0 and M1 cells did not significantly alter the CCI-induced mechanical hypersensitivity (Fig. 4a). None of the macrophage populations modified CCI-induced heat hypersensitivity (Fig. 4b) or mechanical thresholds (Fig. 4c) and heat latencies (Fig. 4d) in sham-operated animals.

### M2 macrophage-induced analgesia is mediated by opioids

Since M2 macrophages secreted opioid peptides in vitro (Fig. 3), we next asked whether opioids contribute to the M2-mediated amelioration of mechanical hypersensitivity in vivo. To determine this, we used NLXM, a peripherally restricted opioid receptor antagonist. NLXM (10 μg) was injected at the CCI site, 24 h after the second injection of M2-polarized macrophages. We found that the M2 macrophage-induced analgesic effect was abolished 15 min after injection of NLXM. The M2 cell analgesic effect recovered 1 h after NLXM injection, consistent with a relatively short duration of action of NLXM (Fig. 5). These data demonstrate that the analgesia following perineural M2 macrophage injection was mediated by local opioid receptors.

### Fluorescent tracking of transferred polarized macrophages shows that they remain at the injured nerve

**Fig. 6:**
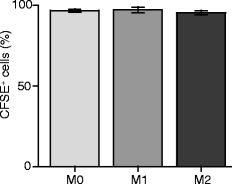
CFSE labeling of cultured macrophages following polarization. CFSE (0.5 μM) was added to cells immediately after polarization and CFSE staining was measured ~24 h later using flow cytometry. *p* > 0.05 (one-way RM ANOVA). Data show the mean ± SEM (*n* = 8 samples per group)

In order to determine whether injected macrophages remain at the nerve, we used the fluorescent tracer CFSE to mark cultured macrophages before injection. Figure 6 shows the percentage of CFSE^+^ cultured cells analyzed using flow cytometry 24 h after CFSE staining. Around 98 % of cells from all treatments were CFSE positive, with no significant differences between M0, M1, and M2 cells. Live/dead staining did not show any difference in viability compared to unstained cells (data not shown).

**Fig. 7:**
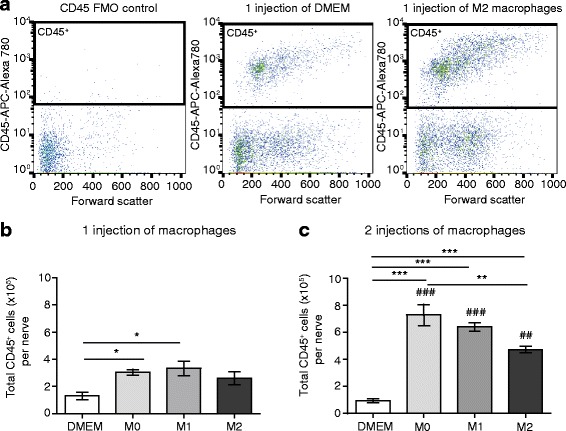
Quantification of CD45^+^ cells in injured nerves following perineural transfer of CFSE-labeled polarized macrophages. **a** Representative *dot blots* of CD45 staining: (*left panel*) CD45 FMO-negative control; (*middle panel*) CD45^+^ cells after one injection of DMEM only; (*right panel*) CD45^+^ cells following one injection of M2 macrophages. **b** Total number of CD45^+^ cells per nerve after one injection of DMEM only or CFSE-labeled M0, M1, and M2 cells. **c** Total number of CD45^+^ cells per nerve after two injections of DMEM only or CFSE-labeled M0, M1, and M2 cells. In all experiments, DMEM- or CFSE-labeled macrophages were injected at the injury site on day 14 (one injection) or on days 14 and 15 (two injections) after CCI. Animals were killed 24 h after the first injection and 24 h after the second injection, and cells from the nerves were analyzed using flow cytometry and FlowJo software. *p < 0.05, **p < 0.01, ***p < 0.001 (one-way ANOVA, Bonferroni’s test); ^##^ *p* < 0.01; ^###^ *p* < 0.001 compared to same treatment 24 h after the first injection (unpaired *t* test). Data show the mean ± SEM (*n* = 6 animals per group)

CFSE-stained M0, M1, and M2 cells (5 × 10^5^ cells each) or control DMEM only were then injected at the injury site on day 14 (one injection) or on days 14 and 15 (two injections) after CCI. Mice were killed 24 h after the first injection and 24 h after the second injection, and cell numbers from the nerves were verified by quantification of CD45^+^ cells and F4/80^+^ macrophages using flow cytometry. Figure 7a shows representative dot blots, demonstrating CD45^+^ cells after the first injection of DMEM or M2 macrophages, with little CD45 staining in the CD45 FMO negative control. Quantitative analysis revealed significantly higher numbers of CD45^+^ cells after the first injection of M0 and M1, but not M2 cells, compared to DMEM control which represents endogenous CD45^+^ cells (Fig. 7b). After the second injection, the number of CD45^+^ cells significantly increased following injection of all three M0, M1, and M2 populations compared to the DMEM group, but there was significantly fewer CD45^+^ cells in animals receiving M2 vs. M0 cells (Fig. 7c). The number of CD45^+^ cells from the M0-, M1-, and M2-injected animals was significantly higher after the second injection compared to the number following the first injection. The CD45^+^ cell numbers in control groups only receiving DMEM did not differ between the two time points (Fig. 7b, c).

**Fig. 8:**
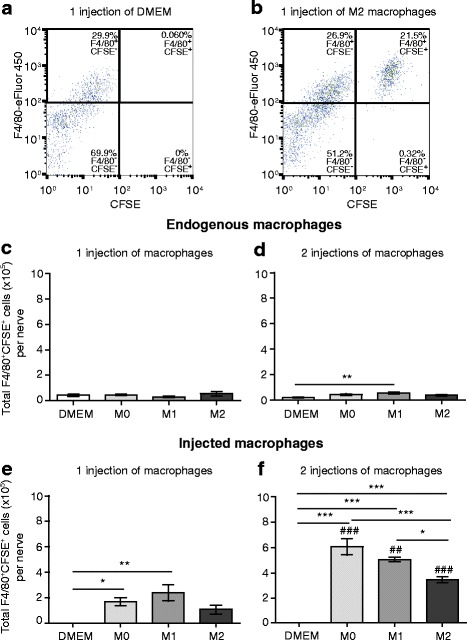
Quantification of endogenous and injected macrophages in injured nerves following perineural transfer of CFSE-labeled polarized macrophages. **a** Representative *dot blot* of F4/80 and CFSE staining after one injection of DMEM only, showing endogenous macrophages (F4/80^+^CFSE^−^) in the *top left quadrant*, and the lack of injected macrophages (F4/80^+^CFSE^+^) in the *top right quadrant*. **b** Representative *dot blot* of F4/80 and CFSE staining after one injection of CFSE-labeled M2 cells, showing endogenous macrophages (F4/80^+^CFSE^−^) in the *top left quadrant* and injected macrophages (F4/80^+^CFSE^+^) in the *top right quadrant*. **c** Total number of endogenous macrophages (F4/80^+^CFSE^−^) after one injection of DMEM alone or CFSE-labeled M0, M1, and M2 cells. **d** Total number of endogenous macrophages (F4/80^+^CFSE^−^) after two injections of DMEM alone or CFSE-labeled M0, M1, and M2 cells. **e** Total number of injected macrophages (F4/80^+^CFSE^+^) after one injection of DMEM alone or CFSE-labeled M0, M1, and M2 cells. **f** Total number of injected macrophages (F4/80^+^CFSE^+^) after two injections of DMEM alone or CFSE-labeled M0, M1, and M2 cells. In all experiments, DMEM- or CFSE-labeled macrophages were injected at the injury site on day 14 (one injection) or on days 14 and 15 (two injections) after CCI. Animals were killed 24 h after the first injection and 24 h after the second injection, and cells from the nerves were analyzed using flow cytometry and FlowJo software. *p < 0.05, **p < 0.01, ***p < 0.001 (one-way ANOVA, Bonferroni’s test); ^##^ *p* < 0.01, ^###^ *p* < 0.001 compared to the same treatment 24 h after the first injection (unpaired *t* test). Data show the mean ± SEM (*n* = 6 animals per group)

Next, we analyzed the F4/80^+^ macrophages in the nerves. As we were able to show that the vast majority of CFSE-stained cultured macrophages remained CFSE positive 24 h after staining (Fig. 6), we were able to conclude that F4/80^+^CFSE^+^ cells were injected macrophages, while F4/80^+^CFSE^-^ cells were endogenous macrophages. This can be seen in the representative dot blots in Fig. 8 showing that after the first perineural injection of DMEM alone, there was virtually no staining for injected macrophages (F4/80^+^CFSE^+^; Fig. 8a, top right quadrant), while there was a clear F4/80^+^CFSE^−^ population of endogenous macrophages (Fig. 8a, top left quadrant). In contrast, after the first perineural injection of CFSE-labeled M2 macrophages, there was a clear F4/80^+^CFSE^−^ endogenous population (Fig. 8b, top left quadrant) and F4/80^+^CFSE^+^ injected population (Fig. 8b, top right quadrant) of macrophages.

The quantitative analysis of endogenous macrophages (F4/80^+^CFSE^−^) isolated from the nerves revealed that their absolute numbers were not significantly altered by perineurally injected M0, M1, and M2 cells as compared to the control DMEM-treated group following the first injection (Fig. 8c). After the second injection, the numbers of endogenous macrophages were also unaltered by the transfer of M0 and M2 cells. The second M1 cell injection resulted in a significant, but very small increase in the number of endogenous macrophages (Fig. 8d). Thus, the endogenous macrophage counts were not substantially changed by injection of polarized macrophages.

Analysis of injected macrophages (F4/80^+^CFSE^+^) isolated from the nerves showed that following the first injection, the absolute numbers of M0 and M1, but not M2 cells, were significantly increased compared to the DMEM-injected group (Fig. 8e). After the second injection, the injected macrophage numbers were significantly elevated in all M0-, M1-, and M2-treated groups compared to the DMEM-injected group. There were also higher numbers of injected macrophages in M0- and M1-treated groups compared to the M2-treated group (Fig. 8f). Additionally, the numbers of injected macrophages were significantly higher after the second compared to the first injection of M0, M1, and M2 cells (Fig. 8e, f). Together, the perineurally injected polarized macrophages could be detected, particularly after the second injection, with more M0 and M1 than M2 cells remaining in the injured nerves.

### Injected macrophages modify CD163 and MHC II expression in endogenous macrophages in the injured nerve

**Fig. 9:**
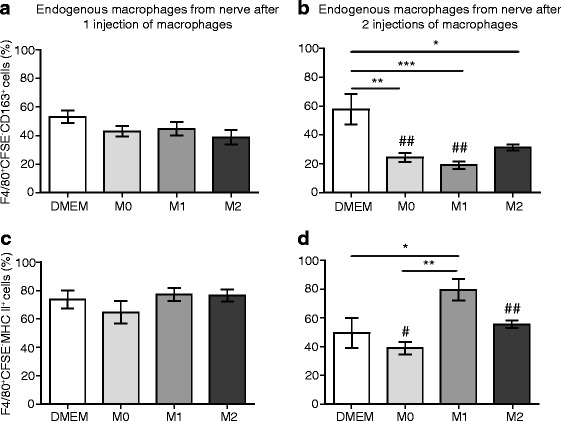
Analysis of CD163 and MHC II expression in endogenous macrophages in injured nerves following perineural transfer of CFSE-labeled polarized macrophages. Endogenous macrophages were identified as F4/80^+^CFSE^−^ cells. **a** Percentage of endogenous macrophages expressing CD163 following one injection of DMEM only or CFSE-labeled M0, M1, and M2 cells. **b** Percentage of endogenous macrophages expressing CD163 following two injections of DMEM only or CFSE-labeled M0, M1, and M2 cells. **c** Percentage of endogenous macrophages expressing MHC II following one injection of DMEM only or CFSE-labeled M0, M1, and M2 cells. **d** Percentage of endogenous macrophages expressing MHC II following two injections of DMEM only or CFSE-labeled M0, M1, and M2 cells. In all experiments, DMEM- or CFSE-labeled macrophages were injected at the injury site on day 14 (one injection) or on days 14 and 15 (two injections) after CCI. Animals were killed 24 h after the first injection and 24 h after the second injection, and cells from the nerves were analyzed using flow cytometry and FlowJo software. *p < 0.05, **p < 0.01, ***p < 0.001 (one-way ANOVA, Bonferroni’s test); ^#^ *p* < 0.05, ^##^ *p* < 0.01 compared to the same treatment 24 h after the first injection (unpaired *t* test). Data show the mean ± SEM (*n* = 6 animals per group)

CFSE staining of injected cells and flow cytometry also allowed us to determine the phenotype of endogenous macrophages using CD163 and MHC II surface marker analysis. We did not find significant differences in the percentage of endogenous macrophages (F4/80^+^CFSE^−^) expressing CD163 after the first injection of polarized M0, M1, or M2 cells in injured nerves (Fig. 9a). After the second injection, however, there was a significant reduction in the percentage of CD163^+^ endogenous macrophages in M0-, M1-, and M2-injected animals compared to DMEM-injected animals (Fig. 9b). Additionally, the percentage of CD163^+^ endogenous macrophages following the second injection of M0 and M1 cells was significantly lower than after the first injection (Fig. 9a, b).

The percentage of endogenous macrophages (F4/80^+^CFSE^-^) expressing MHC II was not significantly altered after the first injection of any polarized cells (Fig. 9c). However, there was a significant increase in the percentage of MHC II^+^ endogenous macrophages following the second injection of M1 cells compared to the DMEM- and M0-injected groups (Fig. 9d). Also, the percentage of MHC II^+^ endogenous macrophages following the second injection of M0 and M2 cells was significantly lower than following the first injection (Fig. 9c, d). These data indicate that the second injection of M0-, M1-, and M2-polarized cells decreased the CD163^+^ population of endogenous macrophages, while M1-injected cells additionally enhanced the MHC II^+^ population of endogenous macrophages.

### Injected macrophages maintain their activation phenotype in the injured nerve

**Fig. 10:**
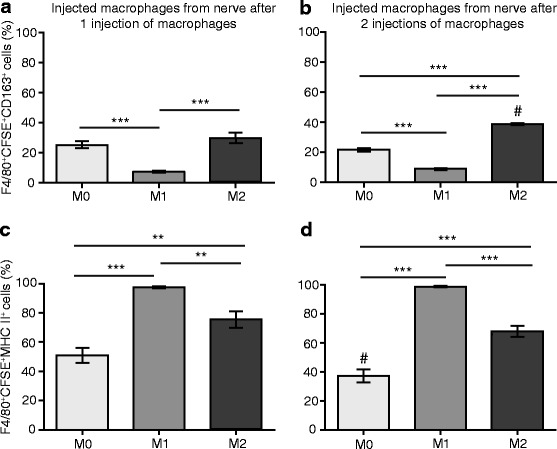
Analysis of CD163 and MHC II expression in perineurally injected CFSE-labeled polarized macrophages in injured nerves. Injected macrophages were identified as F4/80^+^CFSE^+^ cells. **a** Percentage of injected macrophages expressing CD163 following one injection of DMEM only or CFSE-labeled M0, M1, and M2 cells. **b** Percentage of injected macrophages expressing CD163 following one injections of DMEM only or CFSE-labeled M0, M1, and M2 cells. **c** Percentage of injected macrophages expressing MHC II following one injection of DMEM only or CFSE-labeled M0, M1, and M2 cells. **d** Percentage of injected macrophages expressing MHC II following two injections of DMEM only or CFSE-labeled M0, M1, and M2 cells. In all experiments, DMEM medium or CFSE-labeled macrophages were injected at the injury site on day 14 (one injection) or on days 14 and 15 (two injections) after CCI. Animals were killed 24 h after the first injection and 24 h after the second injection, and cells from the nerves were analyzed using flow cytometry and FlowJo software. **p < 0.01, ***p < 0.001 (one-way ANOVA, Bonferroni’s test); ^#^ *p* < 0.05 compared to the same treatment 24 h after the first injection (unpaired *t* test). Data show the mean ± SEM (*n* = 6 animals per group)

Perineurally injected CFSE-stained M0, M1, and M2 cells were analyzed as above for CD163 and MHC II expression following isolation from the nerve. The CD163 expression of injected macrophages (F4/80^+^CFSE^+^) after the first injection showed a similar expression profile to cultured macrophages not stained with CFSE (Fig. 1c), with a significantly higher percentage of CD163^+^ M0 and M2 cells vs. M1 cells (Fig. 10a). This difference was more striking after the second injection when there was also a significantly higher percentage of CD163^+^ M2 vs. M0 cells (Fig. 10b). There was also slightly higher percentage of CD163^+^ M2 cells following the second than the first injection (Fig. 10a, b).

The MHC II expression of injected CFSE-stained macrophages (F4/80^+^CFSE^+^) was also comparable to cultured macrophages not stained with CFSE (Fig. 1d), with a significantly higher percentage of MHC II^+^ M1 vs. M0 and M2 cells, and of MHC II^+^ M2 vs. M0 cells, both after the first (Fig. 10c) and second (Fig. 10d) injections. The percentage of MHC II^+^ M0 cells was slightly lower following the second than the first injection (Fig. 10c, d).

## Discussion

We have shown here that the anti-inflammatory M2-polarized macrophages contain and secrete higher levels of opioid peptides, including Met-enkephalin, dynorphin, and β-endorphin, compared to pro-inflammatory M1 and unstimulated M0 macrophages in vitro. When transferred to the nerve injury site, M2 macrophages, but not M1 and M0 cells, reduced neuropathy-induced tactile hypersensitivity in vivo. The M2 macrophage-induced analgesia was mediated by opioids at the damaged nerves since it was blocked by a local injection of the opioid receptor antagonist naloxone methiodide. None of the injected macrophages affected the sensitivity in sham-operated animals.

Macrophages undergo a process of activation upon disease or injury. The widely described M1/M2 classification vastly simplifies the complexity of in vivo pathological conditions, which often involve a spectrum of macrophage activation states [19, 20, 53]. Nevertheless, despite these limitations, the M1/M2 model provides useful conceptual insights into studying macrophage phenotype-dependent processes [20]. Indeed, polarization using LPS and IFN-γ for M1 and IL-4 for M2 macrophages are established methods and have been successfully employed in many studies [19, 35–37, 54]. Our flow cytometry data, which indicate a higher percentage of MHC II^+^ cells in the M1-polarized population and the predominance of CD163^+^ cells in M2 population, are in line with previous studies [47, 55–57]. Similarly, higher levels of pro-inflammatory cytokines (MCP-1, TNF-α, IL-6, IFN-γ) secreted by M1 than by M2-polarized macrophages are in agreement with earlier reports [54, 57–59]. The M2 phenotype has also been characterized by the production of anti-inflammatory cytokines; however, in our conditions, the extracellular levels of IL-10 were below detection limit. In fact, the published data on the IL-10 secretion are inconsistent. Some studies reported elevated levels of IL-10 released by M2 vs. M0 and M1 cells using IL-4 as the M2 polarizing agent [54], while other authors specifically modified polarizing protocols using a combination of IL-4, IL-10, and transforming growth factor-β, or by adding LPS (which typically is used as M1 polarizing agent) to achieve enhanced IL-10 production by M2 cells [60, 61]. Furthermore, higher levels of IL-10 released by M1 than by IL-4-polarized M2 macrophages were also reported [35, 57]. Thus, it appears that using IL-10 as a marker of macrophage phenotype might be challenging since its measurements can be differentially influenced by methodological factors [62]. Nevertheless, our analysis of the CD163 and MHC II expression and pro-inflammatory cytokine profile is clearly consistent with the M1/M2 polarization status reported in the literature.

It is interesting that M2 macrophages contained and secreted substantially higher levels of the opioid peptides Met-enkephalin, dynorphin, and β-endorphin than M0 and M1 macrophages. The M2 cells contained up to 1.5-fold and released up to 1.8-fold higher opioid levels than M0 cells. The differences were even greater when comparing M2 with M1 cells: M2 cells contained up to 5.8-fold more and released up to 3.6-fold higher opioid levels than M1 cells. Importantly, these effects are of relevance in vivo because the transfer of M2 macrophages at the injured nerve diminished CCI-induced mechanical hypersensitivity. This analgesia was dependent on opioids, as demonstrated by its complete blockade by the opioid receptor antagonist naloxone methiodide. These actions were specific to neuropathic pain since M2 macrophages were ineffective following application at the sham-operated nerves. Although some improvement of neuropathic pain by M0 cells could be anticipated, the lower capacity to release opioids might account for their ineffectiveness. It is perhaps more surprising that M1 macrophages did not enhance mechanical or heat sensitivity, despite substantially lower released amounts of opioid peptides and higher levels of pro-inflammatory cytokines compared to M0 and M2 cells.

In order to therefore gain greater insight into the characteristics of the polarized macrophages transferred perineurally, we labeled them using CFSE as a fluorescent marker before injection and carried out flow cytometry analysis of all cells isolated from injured nerves ex vivo. The CFSE labeling enabled us to distinguish injected (CFSE^+^) from endogenous (CFSE^−^) macrophages. We examined the cells 24 h after the first injection and 24 h after the second injection, according to in vivo pain/analgesia testing. Our quantitative analysis revealed that injected polarized macrophages could be detected in the nerve. Thus, the total number of injected macrophages (F4/80^+^CFSE^+^) was significantly increased particularly after the second injection of M0, M1, and M2 cells compared to DMEM application, while the number of endogenous macrophages (F4/80^+^CFSE^−^) was not substantially altered across the treatments. This finding also suggests that the equivalent increase in the total number of all leukocytes (CD45^+^ cells) in the nerve was brought about by the injected macrophages. However, the numbers of injected cells isolated from nerves were lower than expected, since we injected 5 × 10^5^ macrophages twice, but found around 5–6 × 10^5^ of M0 and M1 cells and about 4 × 10^5^ of M2 cells after the second injection. This might be related to technical reasons since isolation of cells from the nerve 15 and 16 days following CCI is difficult due to a buildup of scar tissue, which can lead to loss of cells. Another possibility is that some injected cells were removed/phagocytosed by endogenous cells, and it remains to be clarified why M2 cells were the most affected. Analysis of the surface marker profile of the injected (CFSE^+^) M0, M1, and M2 macrophages following perineural injection showed that these cells retained their phenotype in vivo; their CD163 and MHC II expression at both time points following transfer closely resembled the CD163 and MHC II expression profiles seen in our in vitro culture experiments. However, the phenotype of endogenous (CFSE^−^) macrophages showed some modifications. Although their CD163 and MHC II expression profile was not altered following the first injection of M0, M1, and M2 cells, the second injection of these cells resulted in a decreased percentage of endogenous macrophages expressing CD163 when compared to the DMEM control. In contrast, the percentage of MHC II^+^ endogenous macrophages was higher following the second injection of M1 cells, with no significant alterations induced by injected M0 and M2 macrophages compared to the DMEM control. Taken together, these results demonstrate that a fraction of injected polarized macrophages remain at the injured nerves and generally preserve their original phenotype. Additionally, the injected cells seem to have an effect on the endogenous macrophages, with injected M1 cells appearing to increase the M1 phenotype of endogenous macrophages based on the elevated MHC II profile. The effect on CD163 expression is less clear since it was decreased by all injected cells irrespective of their phenotype, and therefore, the data must be interpreted cautiously as characterization of macrophage phenotypes in vivo is challenging and analysis of a broad spectrum of markers would be required [19, 20, 53].

Recapitulating the findings of our in vitro, in vivo and ex vivo experiments, it can be concluded that the ability of M2 cells to produce and secrete the highest levels of opioid peptides and to preserve their M2 phenotype following perineural transfer underlie their analgesic effects in vivo. The fact that their numbers ex vivo were lower than expected may explain the observation that the neuropathy-induced mechanical hypersensitivity was not completely reversed to the pre-injury thresholds. There is also a clear agreement between in vivo and ex vivo experiments showing that two M2 cell injections were more effective than the single one. The need for multiple macrophage injections was also demonstrated for recovery from spinal cord injury [63]. Although the numbers of M0 cells were higher compared to M2 cells following the perineural transfer, the finding that they released lower amounts of opioid peptides may account for their inability to attenuate neuropathy-induced hypersensitivity. On the other hand, the M1 cells did not enhance sensitivity following either CCI or sham operation, despite the fact that they secreted the lowest amounts of opioid peptides and the highest levels of pro-inflammatory cytokines, preserved their M1 phenotype following perineural injection and seemed to enhance the M1 phenotype of endogenous macrophages, and were detected ex vivo in higher numbers than M2 cells. Though somewhat unexpected, the findings regarding M0 and M1 cells are in line with earlier studies suggesting a limited role of macrophages in the generation of pain. Hence, a general depletion of macrophages often resulted either in moderate elevations or no changes in the nociceptive thresholds in models of neuropathic, inflammatory, and postoperative pain [15–17, 64, 65]. Furthermore, injection at the nerve or into a hind paw of non-polarized peritoneal or bone marrow-derived macrophages or of IFN-γ-stimulated macrophages did not induce pain in sham-operated animals and did not enhance hypersensitivity induced by neuropathy or paw incision [17, 22, 27]. Although Abbadie et al. [66] reported decreased macrophage infiltration and amelioration of neuropathic pain in mice lacking C-C chemokine receptor 2, indicating pain-promoting actions of macrophages, a recent study found increased numbers of neutrophils as a compensatory change in these mice [67], which makes the data interpretation difficult since neutrophils can decrease hypersensitivity via opioid peptides [68, 69]. In fact, the paper by Ghasemlou et al. [67] suggested that not pro-inflammatory monocytes, but subset(s) of CD11^+^ myeloid cells (e.g., dendritic, Langerhans, natural killer, and/or mast cells) may mediate postoperative and inflammatory hypersensitivity. Together, the role of macrophages, including pro-inflammatory M1 cells as drivers of pain might be smaller than generally assumed, and the investigation of a relative contribution of macrophages vs. other cells such as fibroblasts, endothelial, and Schwann cells is warranted [70].

In contrast, our findings highlight the favorable actions of M2 macrophages in neuropathic pain. In previous studies, macrophage depletion enhanced pain due to impaired actions of opioid peptides or IL-10 in the CFA and carrageenan inflammatory pain models, respectively [25, 71]. The analgesic effects of anti-inflammatory cytokines derived from M2 cells in response to a PPAR-γ agonist or IL-4 application have been suggested by showing a correlation between the attenuation of pain and enhanced mRNA levels of IL-10 in postoperative and neuropathic pain models; the direct contribution of IL-10, however, has not been examined [22, 27, 28]. Additionally, promoting the anti-inflammatory macrophage phenotype in response to PPAR-γ activation resulted in naloxone-reversible attenuation of carrageenan or CFA-induced inflammatory pain [23, 24]. Thus, accumulating evidence demonstrates the beneficial actions of macrophages in pain control, and our findings define the M2 phenotype as the most relevant macrophage source of opioid peptides to attenuate neuropathy-induced mechanical hypersensitivity.

Interestingly, in our experiments, M2 macrophages did not ameliorate heat hypersensitivity resulting from neuropathy. Few studies reported attenuation of both mechanical and heat hypersensitivity by activation of macrophage PPAR-γ or toll-like receptor 4 in incisional and CFA-induced inflammatory pain models [22, 25]. Several other reports, however, found that a phenotypic shift to M2 in response to a PPAR-γ agonist or morphine did not improve heat, but attenuated mechanical hypersensitivity following hind paw incision or inflammation induced by carrageenan or CFA [23, 24, 65]. While the reasons for the differences among these studies and the mechanisms underlying the role of immune cells in the modulation of different pain modalities remain to be elucidated [67], our findings add to those which suggest that promoting an M2 phenotype might be particularly beneficial in improving mechanical hypersensitivity.

Although we focused on analgesic actions of in vitro polarized, and in vivo transferred M2 macrophages, the role of endogenous M2 macrophages should also be addressed. Our data indicate that endogenous M2 macrophages do not substantially contribute to the constitutive control of neuropathic pain. Hence, although endogenous M2 cells were present in injured nerves (DMEM groups in Fig. 9a, b), mice experienced substantial mechanical and heat hypersensitivity following CCI in vivo. Our earlier studies suggest that endogenous immune cells which accumulate at the injured nerve do contain opioid peptides, but need to be stimulated to release them and ameliorate neuropathic pain in animals, for example, by corticotropin-releasing factor or opioid receptor agonists [11, 14, 29, 30]. These endogenous cells included macrophages, but with previous approaches, it was not possible to distinguish their phenotypes. Thus, it is conceivable that endogenous macrophages need to be activated/polarized toward M2 phenotype in vivo to exert opioid-mediated analgesia, which would constitute a separate project.

## Conclusions

As we have employed the macrophage transfer as a tool to delineate their pain-modulating properties, other researches have utilized this strategy as a therapy for nervous system disorders such as spinal cord injury [72, 73]. Since the injection of macrophages might result in their own [37], or endogenous cell phenotypic changes (this study), future work will be needed to verify the utility of the adoptive cell transfer in improving the disease outcome. Our data suggest that macrophages might be less relevant to the generation of pain since polarizing these cells to an M1 pro-inflammatory phenotype did not exacerbate mechanical or heat sensitivity. In fact, our findings emphasize the need to focus on the production of M2-inducing therapeutics. For the first time, we have provided robust analysis of opioid peptide content and release from M0, M1, and M2 macrophages. We have shown that M2 cells can be manipulated in vitro to produce an opioid-mediated analgesic effect in neuropathy in vivo. The next step will be to define the most effective strategy to promote the opioid-mediated M2 responses for the better control of pathological pain.

## Abbreviations

ANOVA: Analysis of variance
CBA: Cytokine bead array
CCI: Chronic constriction injury
CD: Cluster of differentiation
CFA: Complete Freund’s adjuvant
CFSE: Carboxyfluorescein succinimidyl
DMEM: Dulbecco’s modified Eagle medium
Dynorphin: Dynorphin A (1-17)
EIA: Enzyme immunoassay
FMO: Fluorescence minus one
HBSS: Hanks’ balanced salt solution
IFN-γ: Interferon-γ
IL: Interleukin
LPS: Lipopolysaccharide
MCP-1: Monocyte chemoattractant protein-1
MCSF: Macrophage colony-stimulating factor
MHC II: Major histocompatibility complex II
NLXM: Naloxone methiodide
PPAR-γ: Peroxisome proliferator-activated receptor-γ
RIA: Radioimmunoassay
RM: Repeated measures
TNF-α: Tumor necrosis factor-α
